# Association between Incidental Pelvic Inflammation and Aggressive Prostate Cancer

**DOI:** 10.3390/cancers14112734

**Published:** 2022-05-31

**Authors:** Dimple Chakravarty, Parita Ratnani, Li Huang, Zachary Dovey, Stanislaw Sobotka, Roy Berryhill, Harri Merisaari, Majd Al Shaarani, Richa Rai, Ivan Jambor, Kamlesh K. Yadav, Sandeep Mittan, Sneha Parekh, Julia Kodysh, Vinayak Wagaskar, Rachel Brody, Carlos Cordon-Cardo, Dmitry Rykunov, Boris Reva, Elai Davicioni, Peter Wiklund, Nina Bhardwaj, Sujit S. Nair, Ashutosh K. Tewari

**Affiliations:** 1 Department of Urology, Icahn School of Medicine at Mount Sinai, New York, NY 10029, USA; parita.ratnani@mountsinai.edu (P.R.); zachary.dovey@mountsinai.org (Z.D.); stanislaw.sobotka@mountsinai.org (S.S.); roy.berryhill@mountsinai.org (R.B.); kamlesh.yadav@tamu.edu (K.K.Y.); sneha.parekh@mountsinai.org (S.P.); vinayak.wagaskar@mountsinai.org (V.W.); peter.wiklund@mountsinai.org (P.W.); nina.bhardwaj@mssm.edu (N.B.); sujit.nair@mountsinai.org (S.S.N.); ash.tewari@mountsinai.org (A.K.T.); 2 Department of Urology, Sun Yat-Sen Memorial Hospital, Sun Yat-Sen University, Guangzhou 510275, China; huangli26@mail.sysu.edu.cn; 3 Department of Radiology, University of Turku, 20014 Turku, Finland; haanme@utu.fi (H.M.); ivjamb@utu.fi (I.J.); 4 Medical Imaging Centre of Southwest Finland, Turku University Hospital, 20521 Turku, Finland; 5 Department of Pathology, Icahn School of Medicine at Mount Sinai, New York, NY 10029, USA; malshaarani@mfa.gwu.edu (M.A.S.); rachel.brody@mountsinai.org (R.B.); carlos.cordon-cardo@mssm.edu (C.C.-C.); 6 Department of Pathology, George Washington University Hospital, Washington, DC 20037, USA; 7 Department of Hematology & Medical Oncology, Icahn School of Medicine at Mount Sinai, New York, NY 10029, USA; richa.rai@mssm.edu; 8 School of Engineering Medicine, Texas A&M University, Houston, TX 77030, USA; 9 Division of Cardiovascular Research, Albert Einstein College of Medicine, New York, NY 10467, USA; sandeep.mittan@mountsinai.org; 10 Department of Genetics and Genomic Sciences, Icahn School of Medicine at Mount Sinai, New York, NY 10029, USA; yulia.kodysh@mountsinai.org (J.K.); dmitry.rykunov@mssm.edu (D.R.); boris.reva@mssm.edu (B.R.); 11 Decipher Biosciences, A Subsidiary of Veracyte Inc., South San Francisco, CA 94080, USA; elai.davicioni@veracyte.com; 12 Department of Hematology and Oncology, Icahn School of Medicine at Mount Sinai, New York, NY 10029, USA

**Keywords:** PSA (prostate specific antigen), PSAD (prostate specific antigen density), PCa (prostate cancer), MRI (magnetic resonance imaging), PI-RADS (prostate imaging reporting and data system version 2), ECE (extracapsular extension), EPE (extra prostatic extension), PCa (prostate cancer), BCR (biochemical recurrence), RALP (robot-assisted laparoscopic prostatectomy), AP (adverse pathology), EMT (epithelial-to-mesenchymal transition), DDR (DNA damage and repair), IL (interleukin)

## Abstract

**Simple Summary:**

This study reports a significant association of pelvic inflammation with prostate cancer (PCa) aggressiveness in a large cohort of men undergoing robot-assisted laparoscopic prostatectomy (RALP) for localized PCa. In addition, PCa patients with pelvic inflammation had elevated expression of inflammation-associated genes and cancer-driving pathways in their tumors. Increased systemic inflammation with activation of the IL6-STAT pathway was seen in prostate cancer patients with pelvic inflammation. The presence of pelvic inflammation in prostate cancer patients suggests aggressive disease with a potential to develop biochemical recurrence and metastasis. This study is highly relevant as it allows us to follow prostate cancer patients with pelvic inflammation for metastasis closely. It also suggests that inhibiting the STAT-IL6 pathway would benefit these patients.

**Abstract:**

The impact of pelvic inflammation on prostate cancer (PCa) biology and aggressive phenotype has never been studied. Our study objective was to evaluate the role of pelvic inflammation on PCa aggressiveness and its association with clinical outcomes in patients following radical prostatectomy (RP). This study has been conducted on a retrospective single-institutional consecutive cohort of 2278 patients who underwent robot-assisted laparoscopic prostatectomy (RALP) between 01/2013 and 10/2019. Data from 2085 patients were analyzed to study the association between pelvic inflammation and adverse pathology (AP), defined as Gleason Grade Group (GGG) > 2 and ≥ pT3 stage, at resection. In a subset of 1997 patients, the association between pelvic inflammation and biochemical recurrence (BCR) was studied. Alteration in tumor transcriptome and inflammatory markers in patients with and without pelvic inflammation were studied using microarray analysis, immunohistochemistry, and culture supernatants derived from inflamed sites used in functional assays. Changes in blood inflammatory markers in the study cohort were analyzed by O-link. In univariate analyses, pelvic inflammation emerged as a significant predictor of AP. Multivariate cox proportional-hazards regression analyses showed that high pelvic inflammation with pT3 stage and positive surgical margins significantly affected the time to BCR (*p* ≤ 0.05). PCa patients with high inflammation had elevated levels of pro-inflammatory cytokines in their tissues and in blood. Genes involved in epithelial-to-mesenchymal transition (EMT) and DNA damage response were upregulated in patients with pelvic inflammation. Attenuation of STAT and IL-6 signaling decreased tumor driving properties of conditioned medium from inflamed sites. Pelvic inflammation exacerbates the progression of prostate cancer and drives an aggressive phenotype.

## 1. Introduction

Multiple factors contribute to the complex biological phenotype of prostate cancer (PCa), and emerging data suggest that inflammation is a driver of PCa [1,2,3]. Mechanistically, pro-inflammatory cytokines and chemokines provoke adverse tumor biology that facilitates cancer development by turning the tumor microenvironment (TME) from friend to foe [1,2,3]. The inflammatory milieu within the prostate is linked with the activation of inflammation-associated genes (intrinsic pathways) or due to the anatomic proximity to local inflammation (extrinsic pathways, e.g., from colon, rectum, pelvic fat, or connective tissue). While the cancer-promoting effects of inflammation on PCa is likely agnostic to its origin [4], the role of pelvic inflammation on PCa progression has not been previously investigated. This study center’s busy robot-assisted laparoscopic prostatectomy (RALP) program provided a unique opportunity to study pelvic inflammation (Figure S1) and association with cancer. We found that incidental pelvic inflammation was more often associated with aggressive PCa on final pathology. Thus, we hypothesized that pelvic inflammation promotes biologically aggressive disease in PCa patients. Accordingly, the goals of the study are: (i) epidemiological and observational research documenting details of pelvic inflammation as observed during RALP and studying its association with adverse pathology (AP) on final pathology and future biochemical recurrence (BCR); (ii) evaluation of pelvic inflammation with systemic cytokine response; (iii) analysis of inflammatory pelvic tissue and paracrine association with PCa; (iv) investigation of tumor transcriptome and tumor microenvironment; and (v) in vitro functional assays to test the effect of inflamed pelvic tissues on cancer properties of PCa.

## 2. Materials and Methods

### 2.1. Study Population

Retrospective analysis was performed at a single center, Mount Sinai Hospital, New York, USA, using a cohort of 2278 men with prostate cancer who underwent RALP from January 2013 to October 2019, after institutional review board (IRB) approval. After excluding patients with missing clinical information (*n* = 193) and those who underwent simple prostatectomy (*n* = 2), a total of 2085 patients were eligible for study (Figure S2).

A subgroup analysis to study prediction of biochemical recurrence (BCR) was performed in patients with at least three-year follow-up. In total, 1997 (96%, 1997/2085) were included in this sub-analysis; of those, 510 developed BCR, defined as two consecutive PSA values higher than 0.2 ng/mL and rising (Figure S3).

### 2.2. Variables and Outcome

Baseline clinical and pathological data included age, prostate specific antigen (PSA), multiparametric magnetic resonance imaging (mpMRI) prostate volume, race, extracapsular extension (ECE) on MRI, biopsy Gleason, lymph vascular invasion (LVI), extra prostatic extension, positive surgical margin, and seminal vesicular invasion (SVI).

#### 2.2.1. Definition of Adverse Pathology

Adverse pathology (AP) [5] was defined as T3 or higher TNM (tumor nodes metastasis) staging system, and/or final Gleason Group > 2 on post-surgical histopathology. The patients were subsequently divided into two groups according to presence or absence of AP features for which descriptive statistics was performed.

#### 2.2.2. Definition of Biochemical Recurrence (BCR)

Biochemical recurrence was defined as an elevated prostate-specific antigen (PSA) > 0.2 ng/mL in two consecutive measurements after RALP, indicative of treatment failure [6]. Persistent PSA was defined as a PSA ≥ 0.1 ng/mL at 6–8 weeks after RP [7]. Since patients with PSA persistence at 6–8 weeks also have a high risk of PCa recurrence, they have been grouped with BCR patients in the analysis. Overall, the prediction model included all pre- and post-operative variables.

#### 2.2.3. Grading Pelvic Inflammation

Pelvic inflammation observed during RALP (Figure S1) was reviewed and scored by two independent clinicians in a blinded manner and recorded as low (1,2) and high inflammation (3,4). The scoring system was devised to be simple, to limit bias and inter-rater variability, and agreed prior to embarking on scoring based on degree of adhesions and distortion of pelvic anatomy.

In the cohort of 2223 patients, we analyzed agreement between two independent raters to evaluate pelvic inflammation observed during RALP on high-resolution surgery videos. Patients’ pelvic inflammation was scored and recorded using an ordinal scale (0 vs. 1 vs. 2 vs. 3).

Weighted Cohen’s Kappa showed very good inter-rater agreement in the level of inflammation (estimate = 0.928, standard error = 0.0051, 95% confidence limits: 0.918–0.938, *p* < 0.0001). SAS 9.4 software was used in data analysis.

### 2.3. Statistical Analysis

Univariate Analysis was done by chi-square/Fisher’s exact tests for categorical data and Mann–Whitney test for continuous variables. Continuous variables were reported as median and interquartile range (IQR), and categorical variables were reported as frequency (details in Supplementary Methods).

Multivariable logistic regression was used to predict BCR in patients with at least three years of follow-up. Time-to-event outcomes were analyzed using Kaplan–Meier survival curves for patients with low and high pelvic inflammation scores and log rank tests. Cox proportional hazard regression was used to investigate the effect of pelvic inflammation on BCR.

### 2.4. Cell Culture

22RV1 cells were obtained from ATCC and maintained in RPMI + 10% FBS. Primary PCa cells were freshly isolated from prostate tissues post-surgery. PCa explant cultures were performed as described earlier [8,9,10,11,12,13] with modifications (Supplementary Methods). Suspension cells developed using this method was used further for invasion and migration assays. All cell lines have been authenticated by short tandem repeat (STR) profiling and Mycoplasma screened by a PCR-based approach (abmgoods Cat. No. G238).

#### Invasion and Migration Assays

Invasion or migration assays were performed using Millipore Kit using manufacturer’s instructions (Supplementary Methods).

### 2.5. Quantitative RT PCR

Quantitative real-time PCR was performed on cDNA isolated from inflamed and non-inflamed peritoneum, as described earlier.

### 2.6. Microarray Data Analysis

Formalin-fixed paraffin-embedded blocks from RP specimens were submitted for Decipher testing. RNA was extracted and hybridized to Human Exon 1.0 ST microarrays (Thermo-Fisher, Carlsbad, CA, USA), as described previously [14]. Microarray quality control was performed using Affymetrix Power Tools, as described previously [15]. RNA profiles of each sample were computed after probe-set summarization and normalization [16] (Supplementary Methods).

#### Immunohistochemical Analysis of Prostate Tumor Tissues

Next, 3 μm FFPE tissues from RP samples were deparaffinized and probed with antibodies against anti-IL-6 (Proteintech Inc, Rosemont, IL, USA), Ki-67 (Ventana Systems Inc., Harvard, MA, USA) and Vimentin (Roche, Branchburg, NJ, USA), as described previously [17,18]. The staining was scored by a pathologist and blinded scores were recorded.

### 2.7. O-Link

The serum samples from the patients were analyzed using O-link multiplex assay platform with Immuno-oncology panel (O-link Bioscience, Uppsala, Sweden), according to the manufacturer’s instructions. The oncology panel includes 92 proteins associated with immune response (Supplementary Methods).

## 3. Results

### 3.1. Association between Pelvic Inflammation and Adverse Pathology

An association of pelvic inflammation with AP was determined in 2085 men who had RALP (Consort diagram; Figure S2). AP was reported in 43.98% (*n* = 917/2085) patients. The patient’s baseline characteristics are summarized in Table 1.

Inguinal mesh triggers a long-term inflammatory response [19], resulting in local cytokine release, potentially influencing prostate biology. Therefore, to eliminate the confounding effects of hernia mesh, analysis was performed in patients with pelvic inflammation with or without hernia mesh. In univariate analysis, pelvic inflammation (without hernia mesh; *p* ≤ 0.0002) was a strong predictor of AP. Additionally, age, PSA, mpMRI, ECE on MRI, and biopsy (GGG) emerged as significant predictors of AP (*p* ≤ 0.0001) (Table 2). We also collected information on other cofactors of inflammation, e.g., history of diverticulitis, prior abdominal surgeries, etc., but we had very few cases to include in our analysis.

High biopsy Gleason grade group (GGG) has been shown to be significantly associated with AP [20,21]. In multivariate analysis, biopsy GGG (4 or 5), PSA, and pelvic inflammation (with and without hernia mesh) predicted AP (*p* ≤ 0.05) (Table 2).

**Figure 1:**
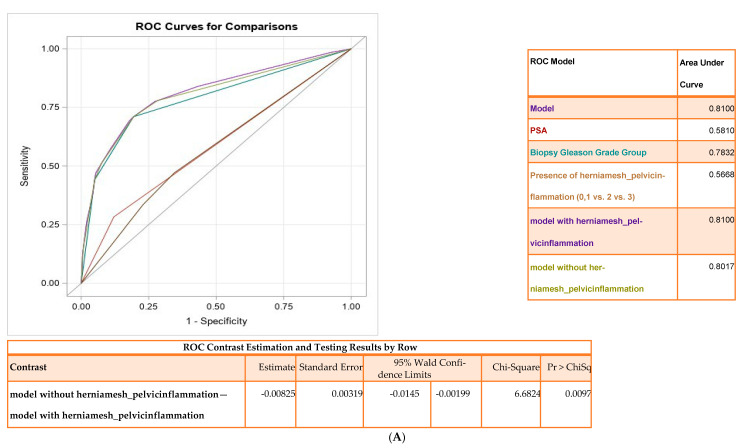

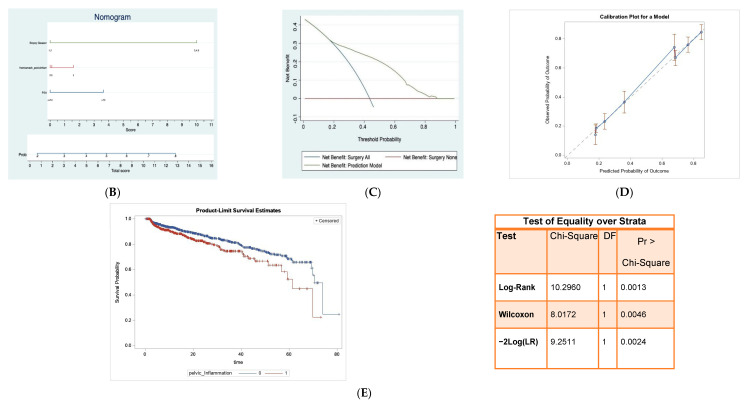
Pelvic inflammation is associated with higher rates of adverse pathology and biochemical recurrence. (**A**) Area under the receiver operating characteristics (ROC) curve analysis comparing the base model with PSA, biopsy Gleason grade group, hernia mesh pelvic inflammation in predicting adverse pathology. (**B**) Nomogram built for the prediction of adverse pathology in the internal cohort. PSA, biopsy Gleason grade group, and pelvic inflammation were significant contributors to the total score demonstrating the probability of AP in the nomogram. (**C**) Decision curve analysis for predicting AP using prediction model. The graph gives the expected net benefit per patient. The unit is the benefit associated with one PCa patient duly undergoing surgery. DCA demonstrates net benefit between the threshold probabilities of ~18% and 88% for model predicting AP. (**D**) The decile calibration plot is the Hosmer–Lemeshow goodness-of-fit test for the logistic regression model. The subjects are divided in 10 groups by using the deciles of the predicted probability of the fitted logistic model. There is good agreement between model’s predicted probability to the empirical probability. (**E**). Kaplan–Meier curve for biochemical recurrence-free survival by pelvic inflammation. Red line illustrates high pelvic inflammation and blue line for low inflammation. Survival curve differences were evaluated using log rank test (*p* = 0.0013).

The AUC for this model was 0.81 (Figure 1A), and pelvic inflammation significantly contributed to the model (*p* = 0.0097). A nomogram (Figure 1B) and decision curve analysis (DCA) highlights the clinical utility of the model with net benefit 18–85% (Figure 1C). Internal validation was performed comparing the mean predicted probability to mean observed outcome (Figure 1D). Since the presence of pelvic inflammation without hernia mesh was found to be a better predictor of AP, we repeated the multivariate analysis in a reduced cohort of 1858 patients after excluding patients with hernia mesh (Table S1). High pelvic inflammation (without hernia mesh) along with PSA and biopsy Gleason grade were significant predictors of AP (AUC of 0.78, *p* < 0.05 (Figure S4). The comparison of models with and without inflammation in the absence of a hernia was statistically significant (*p* = 0.0162). The nomogram and decision curve analysis (DCA) highlights the net benefit of the model (~18% and 96%) (Figure S5A,B).

Multivariate analysis with a backward elimination scheme, in a subgroup of patients in high GGGs (*n* = 881), showed that pelvic inflammation, PSA, and ECE on MRI are significant predictors of AP (all *p* < 0.01; Table S2). The AUC for this model was 0.65 (Figure S6), and the model with and without inflammation was significant (*p* = 0.047). In a separate univariate analysis of patients with pelvic inflammation irrespective of their hernia mesh status, pelvic inflammation, age, PSA, MRI ECE, mpMRI PI-RADS and biopsy GGG emerged as significant predictors of AP with a *p* < 0.0001 (Table S3). On multivariate analysis with backward elimination scheme high pelvic inflammation, age, PSA, and prostate volume on MRI were statistically significant predictors of AP (*p* < 0.001) (Table S3). The AUC for this model was 0.68 (Figure S7).

We next tested the influence of pelvic inflammation on biochemical recurrence and PSA persistence in a cohort of 1997 men with follow-up PSA data (median 16.72 months (7.34, 35.55) months) post-RALP (Figure S3). BCR and PSA persistence was reported in 13.92% (*n* = 278) of patients. The overall median (IQR) age at the time of RP, and preoperative PSA for those with or without BCR and PSA persistence was 65.0 (60.2–69.2) years and 63.0 years (57.1–68.0), and 8.5 (5.6, 16.0) and 5.5 (4.4, 8.2), respectively. The overall median (IQR) follow-up time for patients with BCR and PSA persistence was 44.38 months (25.30, 62.25). Patient’s baseline characteristics (Table S4) and Kaplan–Meier curves demonstrate that patients with high inflammation had earlier time to recurrence (Figure 1E). Survival curve differences between low and high inflammation were evaluated using log rank test, (*p* = 0.0013). The prostate cancer survival probability at the beginning of follow up after RALP until 6.6 years is demonstrated in Figure S8. Univariate proportional hazard models (Table S5) showed high pelvic inflammation was significantly associated with BCR. Additionally, age, PSA, ECE on MRI, high PIRADs score, biopsy GGG (3,4,5), final pathology GGG (3,4,5), PSM, LNI, SVI, and ECE (*p* ≤ 0.05) were significantly associated with BCR. In multivariate cox proportional-hazards regression analyses, high pelvic inflammation (HR: 1.29; 95% CI, 1.01–1.64; *p* = 0.0433), pT3 stage (HR: 3.94; 95% CI, 3.07–5.05; *p* ≤ 0.0001) and PSM (HR: 1.40, 95% CI, 1.01–1.94; *p* = 0.045) were significantly associated with time to BCR and PSA persistence. Further, high pelvic inflammation (HR: 1.32; 95% CI, 1.03–1.68; *p* = 0.0274), pT3 stage (HR: 3.36 95% CI, 2.62–4.30; *p* = < 0.0001) and PSA (HR: 2.81; 95% CI, 2.81–3.60; *p* ≤ 0.0001) were also significantly associated with time to BCR (Supplementary Table S6).

### 3.2. Prostate Cancer Patients with Pelvic Inflammation Demonstrate Elevated Levels of Pro-Inflammatory Cytokines in the Blood

**Figure 2:**
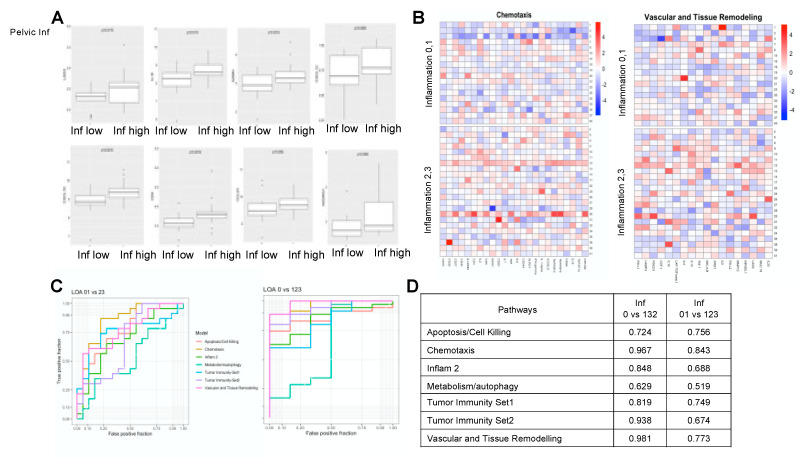
PCa patients with pelvic inflammation demonstrate higher levels of inflammatory markers. (**A**) Box plots indicate expression of key inflammatory modulators LAG3, IL-18, GZMH, CXCL12, CXCL10, CD4, CCL23, and ADGRG1 upregulated in the serum of patients with high inflammation. (**B**) Heat map of differentially regulated genes involved in chemotaxis and vascular and tissue remodeling between low and high inflammation group is shown. (**C**) Association of gene signatures with inflammation was performed and the area under the curve (AUC) of the receiver operating characteristics (ROC) curve determined. (**D**) In pelvic inflammation, 01 vs. 23 comparisons genes associated with chemotaxis had AUC of 0.843, while in inflammation, 01 vs. 123 comparison vascular and tissue remodeling genes had AUC of 0.981.

O-link analysis of serum demonstrated that patients with high inflammation had significantly higher levels of pro-inflammatory chemokines and cytokines (Figure 2A; Supplementary Figure S9). Differentially regulated genes were identified using multivariate multi-class regression models and genes involved in vascular and tissue remodeling, and chemotaxis were found to be significantly different when comparing cases with no inflammation (Inf 0) vs. inflammation (123) (AUC 0.981) or inflammation 0,1(Inf 0,1) vs. inflammation 2,3(Inf 2,3) (AUC 0.843), respectively (Figure 2B–D). LAG3, IL-18, GZMH, CXCL12, CXCL10, CD4, CCL23 and ADGRG1 were differentially regulated in both univariate and multivariate analysis. Supporting evidence from cytokine bead array performed on serum from PCa patients (22 low inf, 22 high inf) further corroborated our findings that pro-inflammatory cytokines are higher in patients with pelvic inflammation (Figure S10).

### 3.3. Inflamed Pelvic Tissue Show Elevated Expression of Genes Involved in the STAT Pathway

**Figure 3:**
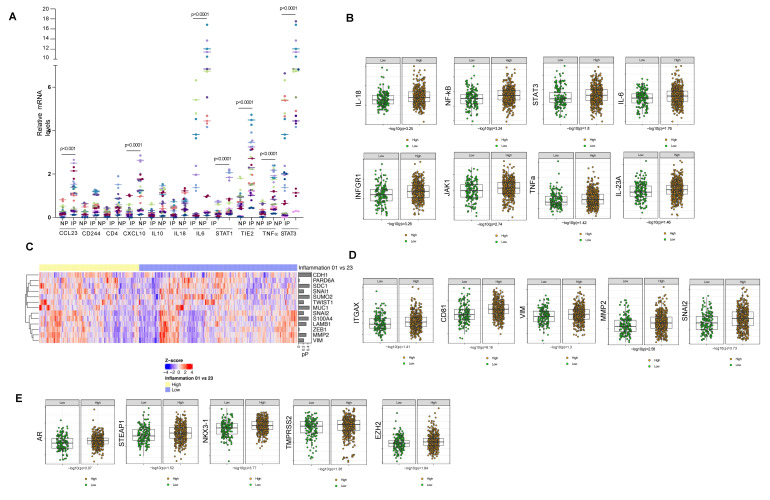
PCa patients with pelvic inflammation demonstrate alteration in inflammation-associated genes at the inflamed site and within the prostate tissue. (**A**) q-PCR analysis of inflamed peritoneum (IP) and non-inflamed control peritoneum (NP) demonstrate upregulation of CCL23, CXCL10, STAT1, STAT3, TIE2 and TNFα. (**B**) Box plots indicate top upregulated genes IL-18, NF-KB, STAT-3, IL-6, INFGR1, JAK1, TNF-α and IL-23A in prostate cancer tumors with incident pelvic inflammation. (**C**,**D**) Heat map shows expression of epithelial-to-mesenchymal transition (EMT) genes in PCa tumors with and without pelvic inflammation (**C**) and box plots of EMT genes are shown in (**D**). (**E**) Box plots of prostate cancer genes upregulated in prostate cancer tumors with incident inflammation is shown.

RNA extracted from inflamed and non-inflamed sites from 22 patients was analyzed using qRT-PCR. We found that CXCL23, CXCL10, IL6, TIE2, TNFα, STAT1, and STAT3 are significantly upregulated in the inflamed peritoneum (Figure 3A) compared to the normal peritoneum. These studies suggest the involvement of the STAT pathway in inflamed pelvic tissue.

### 3.4. Prostate Cancer Patients with Pelvic Inflammation Demonstrate Elevated Levels of Cancer Enabling Transcriptome

We next analyzed the tumor transcriptome of PCa patients with low and high pelvic inflammation evaluated 146 key immune and inflammatory genes [22]. Patients with T3 disease and high pelvic inflammation had significantly higher levels of genes involved in inflammatory response when compared to the patients with T3 disease and low inflammation (Figure S11A). HLA-DRB1, STA1, NR4A1, CD68, STAT3, CNN1, B2M and HLA-DRA were top 10 genes upregulated in T3 disease with high pelvic inflammation (Supplementary Figure S11B). The proinflammatory cytokines significantly upregulated in patients with high pelvic inflammation are seen in Figure 3B and Supplementary Figure S11C. Genes associated with EMT and aggressive prostate cancer were found to be significantly upregulated in the PCa tissues in patients with high pelvic inflammation (Figure 3C,D,E). Interestingly genes involved in the DNA damage repair pathway were also found to be significantly upregulated in the PCa tissue of patients with high pelvic inflammation (Figure S12).

### 3.5. Evidence of Pro-Inflammatory Cytokines in Prostate Tissues of Prostate Cancer Patients with High Pelvic Inflammation

Ki-67 staining revealed a high proliferation index in PCa tissue from patients with high pelvic inflammation (Figure S13A,B). Additionally, levels of IL-6 and Vimentin were elevated in tissues with high inflammation when compared to PCa patients with low inflammation (Figure S13C,D).

### 3.6. Inflamed Pelvic Tissue Contributes to Invasive and Migratory Properties of Prostate Cancer Cells and Can Be Suppressed by Fludarabine and Tocilizumab

**Figure 4:**
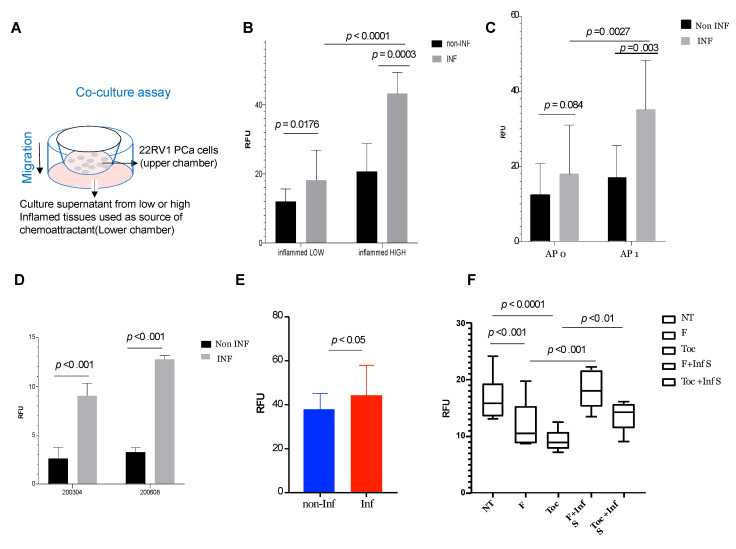
Pelvic inflammation can promote invasion and migration of prostate cancer cell lines and primary prostate cancer cells. (**A**) Schema of migration assay using 22RV1 prostate cancer cell line or primary prostate cells and culture supernatant from inflamed tissues. (**B**) Migration assays using culture supernatants from pelvic tissues of PCa patients stratified into low and high inflammation groups demonstrate that inflammatory mediators from inflamed tissues significantly increases migration of 22RV1 prostate cancer cells. (**C**) Migration assays using culture supernatants from pelvic tissues of PCa patients stratified based on adverse pathology (AP 0 vs. AP 1) demonstrates that inflamed tissues from PCa patients with adverse pathology (AP1) had significantly greater effect on migration of prostate cancer cells when compared to PCa patients without adverse pathology (AP0). (**D**) Effect of pelvic inflammation on autologous primary prostate cancer cells were tested in two PCa patients. Inflamed peritoneum significantly increased the migratory potential of primary prostate cancer cells. (**E**) Invasion assays using culture supernatants from pelvic tissues of PCa patients stratified into low and high inflammation groups demonstrate that inflammatory mediators from inflamed tissues significantly increases invasion of 22RV1 prostate cancer cells. (**F**) As shown in B, inflamed peritoneum increases the migratory potential of prostate cancer cell line 22RV1 and the effects can be blocked by STAT inhibitor fludarabine and IL-6 inhibitor tocilizumab. Interestingly, the effects are reversed by adding supernatant from inflamed tissue.

We explored the direct link between pelvic inflammation and PCa progression in co-culture studies. Inflamed and non-inflamed pelvic tissues were maintained as explant cultures (Figure 4A) and culture supernatants were used for biological assays. We evaluated the effect of culture supernatants on the invasive and migratory properties of 22RV1 PCa cell line and found high invasion and migration inducing characteristics in culture supernatants from inflamed pelvic tissues when compared to non-inflamed pelvic tissues (Figure 4B). Interestingly, culture supernatants derived from inflamed tissues from patients with adverse pathology (AP1) had higher invasive and migratory potential when compared to patients without adverse pathology (AP0) (Figure 4C). We also tested autologous explant cultures and found that supernatants from inflamed peritoneum could also enhance the migration and invasion of autologous primary PCa cells (Figure 4D,E, respectively). As we had found elevated levels of STAT3 and IL-6 in inflamed pelvic tissues, we next tested the effects of blocking STAT pathway and IL-6 pathway. Addition of fludarabine, a STAT1/3 activation inhibitor, or tocilizumab, an IL-6 monoclonal antibody, significantly blocked migration of PCa cells when co-cultured with supernatant from inflamed pelvic tissues. We found that combination of fludarabine and tocilizumab had a stronger inhibitory role on PCa migration when compared to single treatment, suggesting a synergistic effect (Figure 4F). Addition of the supernatant from inflamed pelvic tissues rescued the inhibitory role of the drugs.

## 4. Discussion

Tumor-intrinsic inflammation promotes prostate cancer carcinogenesis. However, the implications of pelvic inflammation and its effect on prostate cancer have never been studied. In this study, we retrospectively investigated the association of pelvic inflammation with PCa aggressiveness and its impact on oncological outcomes in a large cohort of men undergoing RALP for localized PCa. Overall, we observed that pelvic inflammation was a significant predictor of AP in both univariate and multivariate analyses. While several other studies have shown that chronic inflammation within the prostate can facilitate carcinogenesis, pelvic inflammation in this context has not been previously studied. This is the first study to demonstrate that PCa patients with pelvic inflammation are more likely to develop the more aggressive disease. Notably in this cohort, patients underwent surgery but no other cancer-related treatments, thus providing an opportunity to evaluate the direct association of pelvic inflammation with cancer outcomes. The association between pelvic inflammation and aggressive PCa supports the paracrine relationship, involving pro-inflammatory cytokines from inflamed pelvis and prostate tumor cells. PCa patients with high pelvic inflammation had elevated systemic levels of pro-inflammatory cytokines in circulation, and examination of inflamed pelvic tissue taken intraoperatively using quantitative RT-PCR suggested that the systemic pro-inflammatory cytokines detected in the blood were potentially released from these inflamed sites into the systemic circulation. We also show that pelvic inflammation was associated with AP, underscoring the paracrine association’s potential importance between inflamed areas and prostate tumor cells, mediated by pro-inflammatory cytokines. IL-6 and STAT3 pathways play an essential role in PCa progression [3]. Analysis of pelvic inflamed tissues showed evidence of elevated levels of IL-6, STAT3, and IFN genes, suggesting a role for STAT3 signaling. Using IHC analysis, we confirmed high levels of IL-6 and STAT3 observed in inflamed tissues and prostate tissues. In addition, we found that the culture supernatants derived from inflamed tissues potentiated the invasive and migratory behavior in vitro of autologous primary prostate cells or PCa cell lines, which was inhibited by silencing STAT3 and IL-6 signaling using fludarabine, a STAT3 inhibitor, and tocilizumab, an IL-6 inhibitor. This suggests that STAT3 signaling may facilitate the link between pelvic inflammation and PCa aggressiveness. Future work discerning the mechanistic connection is warranted. A deeper examination of the tumor transcriptome in patients with and without pelvic inflammation also demonstrated activation of genes involved in cancer progression as well as those involved in epithelial–mesenchymal transition in the TME, suggesting that pelvic inflammation potentiates pathways of an aggressive phenotype.

Tumor-intrinsic inflammation promotes prostate cancer carcinogenesis. However, the implications of pelvic inflammation and its effect on prostate cancer have never been studied.

Our study results are strengthened by the large cohort size, extensive clinical information, and validations studies. For the first time, our studies demonstrate that pelvic inflammation can drive prostate cancer progression and provides unprecedented knowledge that can advance the field. Future efforts that focus on strategies to identify pelvic inflammation before surgery using improved imaging techniques or blood-based inflammatory markers are critical to identifying PCa patients at high risk. However, our study’s single-center and retrospective nature limit the generalizability of our discovery. Furthermore, our studies lack sufficient follow-up to analyze the impact of pelvic inflammation on oncological outcomes.

Expanded prospective analysis on other multi-institutional cohorts is required to validate pelvic inflammation’s role in prostate cancer progression. In addition, it is critical for a widespread application of our discovery beyond this study’s initial biological conclusions and better management of pelvic inflammation in prostate cancer patients.

## 5. Conclusions

The study’s findings of an association between pelvic inflammation and PCa recurrence raise many interesting questions for future research. Importantly, it does not suggest that pelvic inflammation alone causes PCa; pelvic inflammation exacerbates the progression of the pre-existing disease and may drive an aggressive phenotype. Pelvic inflammation was also associated with upregulation of systemic inflammatory responses and prostate tissue that correlated with upregulation of oncogenic pathways. Thus, although establishing a link between pelvic inflammation and PCa, the mechanisms underlying inflammation’s effect on PCa growth are multifactorial. Based on this study’s findings, the STAT-IL6 pathway may represent one aspect of this complex interaction. However, a deeper understanding of these processes may inform the development of inhibitors of STAT-IL6 pathway signaling to mitigate the effects of inflammation-induced carcinogenesis.

## Figures and Tables

**Table 1:** Baseline characteristics between patients with and without adverse pathology features.

Covariates	Adverse Pathology Absent (*n* = 1168)	Adverse Pathology Present (*n* = 917)	*p* Value
Age (years)	61.08 (55.9, 66.8)	65 (59.4, 69.47)	<0.0001
Surgery_PSA (ng/mL)	5.4 (4.3, 7.2)	6.8 (4.9, 10)	<0.0001
MRI Prostate Volume (mL)	38 (29, 53)	37(28, 51)	0.0213
Pelvic Inflammation			<0.0001
0,1 (Low)	766 (65.58%)	481 (52.45%)	
2,3 (High)	402 (34.42%)	436 (47.55%)	
Race			0.1056
African American	149 (12.76%)	88 (9.60%)	
Caucasian	857 (73.37%)	683 (74.48%)	
Asian	61 (5.22%)	57 (6.22%)	
Others	101 (8.65%)	89 (9.70%)	
MRI PI-RADs			<0.0001
0	41 (3.51%)	14 (1.53%)	
1	66 (5.65%)	39 (4.25%)	
2	132 (11.30%)	50 (5.45%)	
3	121 (10.36%)	42 (4.58%)	
4	556 (47.6%)	330 (35.99%)	
5	252 (21.58%)	442 (48.20%)	
MRI ECE			<0.0001
absent	934 (79.97%)	534 (58.23%)	
present	234 (20.03%)	383 (41.77%)	
Biopsy Gleason group			<0.0001
3 + 3	363 (30.91%)	49 (5.34%)	
3 + 4	583 (49.91%)	215 (23.45%)	
4 + 3	166 (14.21%)	252 (27.48%)	
4 + 4/5 + 3/3 + 5	50 (4.28%)	227 (24.75%)	
4 + 5/5 + 4/5 + 5	8 (0.68%)	174 (18.97%)	

**Table 2:** Univariable and multivariable analysis predicting adverse pathology considering hernia mesh with pelvic inflammation.

Univariable Analysis			Multivariable Analysis	
Covariate	Odds Ratio (5% CI)	*p* Value	Odds Ratio (5% CI)	*p* Value
Age	1.06 (1.05, 1.07)	<0.0001		
PSA				
<10			Ref	
>10.1	2.87 (2.28, 3.62)	<0.0001	2.34 (1.79, 3.06)	<0.0001
Race				
African American	0.69 (0.47, 1.03)	0.0108		
Caucasian	0.97 (0.71, 1.32)	0.6654		
Asian	1.15 (0.72, 1.85)	0.1778		
Others				
MRI Prostate Volume	0.99 (0.99, 1.00)	0.2762		
Herniamesh pelvicinflam				
No inflammation w/o hernia mesh	Ref		Ref	
Inflammation without hernia	1.81 (1.48, 2.22)	0.0002	1.37 (1.08, 1.75)	0.0077
Inflammation with hernia	1.45 (1.10, 1.91)	0.591	0.94 (0.68, 1.31)	0.179
MRI ECE				
Absent	Ref			
present	2.98 (2.44, 3.63)	<0.0001		
MRI PI-RADs		<0.0001		
1,2,3				
4,5	2.43 (1.95, 3.03)			
Biopsy Gleason Grade Group		<0.0001		
1,2	Ref		Ref	
3,4,5	10.19 (8.27, 12.54)		9.49 (0.68, 1.31)	<0.0001

## Data Availability

The data presented in this study are available upon request. The data description is provided in Table 1 and the Supplementary material.

## Supplementary Materials

The following supporting information can be downloaded at: https://www.mdpi.com/article/10.3390/cancers14112734/s1, Figure S1. Pelvic Inflammation (VISUAL GRADING), Figure S2. CONSORT flow diagram of study cohort for Adverse Pathology endpoint, Figure S3. CONSORT flow diagram of study cohort for Biochemical Recurrence and PSA Persistence, Figure S4. Area under receiver operating characteristics for prediction of Adverse Pathology in subgroup considering pelvic inflammation without hernia mesh, Figure S5. A nomogram built for the prediction of Adverse Pathology in the subgroup cohort of 1858 cases, B. Decision curve analysis for predicting AP using prediction model based on subgroup analysis. Figure S6. Area under receiver operating characteristics for prediction of Adverse Pathology considering pelvic inflammation in high biopsy Gleason grade group (3,4,5) in 881 patients, Figure S7: Area under receiver operating characteristics for prediction of Adverse Pathology considering pelvic inflammation, Figure S8: Risk Table: Numbers of survival probability of persons at risk at the beginning of the period of follow-up after Radical Prostatectomy by pelvic inflammation, Figure S9. A. Levels of 92 immune-oncology markers were evaluated in the serum of PCa patients with or without pelvic inflammation using O-link and heat map of differentially expressed genes between the inflammation groups is shown (*p* < 0.05). B. Proteins/cytokines differentially expressed in different pelvic inflammation groups. Figure S10. Proteins/cytokines differentially expressed in different pelvic inflammation groups. Figure S11. Prostate cancer patients with pelvic inflammation demonstrate elevated levels of inflammatory genes. Figure S12. Prostate cancer patients with pelvic inflammation demonstrate elevated levels of DDR genes. Figure S13. Prostate tissues of prostate cancer patients with high pelvic inflammation shows increased proliferation and expression of proinflammatory cytokines. Table S1. Subgroup multivariable analysis predicting adverse pathology considering pelvic inflammation without hernia mesh in 1858 cases, Table S2. Subgroup multivariable analysis predicting adverse pathology in high Biopsy Gleason group (3,4,5) in 881 cases, Table S3. Univariable and Multivariable analysis predicting Adverse Pathology considering pelvic inflammation, Table S4. Baseline characteristics between patients with or without Biochemical Recurrence and PSA persistence features, Table S5. Cox regression models for preoperative and postoperative predictors of BCR and PSA persistence after RP in 1997 patients, Table S6. Cox regression models for predictors including pT3 Stage, pathology stage and pelvic inflammation for predicting BCR after RP in 1997 patients. Ref. [23] cited in Supplementary Materials.
