# Supplementary material for: Association between Incidental Pelvic Inflammation and Aggressive Prostate Cancer
Source: Cancers (Basel). 2022 May 31;14(11):2734. doi: 10.3390/cancers14112734 (PMC9179284; doi:10.3390/cancers14112734)
Supplement: Supplementary file 1 [file cancers-14-02734-s001.zip › cancers-1697081-supplementary.pdf]

Supplementary Methods

Statistical Analysis

Multivariable logistic prediction models were developed to assess the role of pelvic inflammation in providing the individual probability of AP and Cox regression for prediction of BCR, estimated based on patient and tumor characteristics.

The prediction model in Figure 1 included PSA (≤10 ng/mL vs >10.1 ng/mL,) pelvic inflammation score (0 &1 was grouped as low and 2,3 was grouped as high), mpMRI findings (PI-RADS score of 0,1,2,3 was grouped against score of 4,5), biopsy Gleason grade group (1,2 was grouped against score of 3,4,5), final pathology staging (pT2 vs. pT3) and pelvic inflammation with hernia mesh grouped (0,1 (no inflammation w/o hernia mesh vs 2 (inflammation without hernia mesh) vs 3 (inflammation with hernia mesh).

The receiving operator characteristic (ROC) curve was used to estimate how strongly pelvic inflammation can predict Adverse Pathology (AP) at final pathology in univariate and multivariate logistic regression analyses. A nomogram was built based on the coefficients of logit function. Decision Curve Analyses (DCA) was used to evaluate the performance of the prediction model. Internal Validation was performed with a calibration plot to show agreement between predicted probabilities and observed probabilities. For the calibration plots, patients were divided into deciles based on predicted event probability and in each decile averaged probability of predicted events was compared to actual rate of events.

All tests were two-tailed with *p*-value of 0.05 considered statistically significant. SAS 9.4 software (SAS Institute Inc., Cary, North Carolina, USA) and STATA v14 (Stata Corp LLP, College Station, Texas, USA) were used for all statistical analyses.

Microarray data analysis: Transcriptome Analysis

The Decipher assay using Affymetrix platform [14–16] is optimized for use with small amounts of RNA extracted from archived FFPE samples and is the only clinical-grade transcriptome assay for prostate cancer. The Decipher assay was run at the Decipher Biosciences laboratory, located in San Diego, CA, which is certified under the Clinical Laboratory Improvement Amendment (CLIA; 05D2055897), accredited by the College of American Pathologists (CAP, 8859006), and licensed by the New York State Department of Health (NYSDOH, 9018) to run the Decipher FFPE assay.

Multivariable logistic regression was used to identify genes differentially expressed between low and high pelvic inflammation groups.

Primary Prostate cancer cell culture

The procedure to isolate primary prostate cancer cells is compliant with federal regulations for use of clinical biospecimens (45 CFR 46.102(f)). At tissue grossing ~4 mm^3 tissues corresponding to adjacent normal and/or tumor area were freshly dissected by a resident pathologist and de-identified tissue specimens were transported in MACS tissue storage solution (Miltenyi Biotech; catalogue #130-100-008) supplemented with 10µM Rock Inhibitor (Y-27632; Selleck Chemicals; catalogue #S1049), to the laboratory. Tissues were washed twice in DMEM and minced into smaller 1 mm^3 pieces. The cell suspension was prepared using a tumor dissociation kit (Miltenyi Biotech; Catalogue #130-096-334) and gentle-MACS dissociator (program 37C_h_TDK_2) for 60 min. The cell suspension was filtered using a 70µm strainer and collected by centrifugation 430× *g* for 10 min. These cells were then counted and plated for invasion and migration assays.

Invasion and migration assays

Inflamed or normal peritoneum was processed under a laminar flow hood using a sterile technique and rinsed twice in RPM1 medium supplemented with 5% FBS. Tissues were subdivided into approximately 1 mm^3 pieces and placed in a 24-well plate on pre-soaked dental sponges (Novartis #96002) (2–3 pieces per sponge) into 0.5 mL culture media. Plates were placed in an incubator at 37 °C and 5% CO2. The supernatant collected

after 24 h was used as a chemoattractant for invasion and migration assays. The assays were performed as described previously [17] following the manufacturer's instructions.

Quantitative RT PCR

Inflamed and non-inflamed peritoneum was collected during surgery from patients who consented to PPHS/IRB study (Mount Sinai # GCO 14-0318). Total RNA was prepared using Purelink RNA mini kit (Invitrogen) and reverse transcribed using the iscript Advanced cDNA synthesis reagent's following the manufacturer's instructions (BioRad Laboratories, USA). Briefly, tissues were homogenized in lysis buffer to ensure RNAse-free lysis and purified through a mini spin column. Following multiple washes, total RNA was collected into a final volume of 30 μL. Total RNA was measured using nano-drop and 260/230 and 260/280 ratios were considered a measure of quality. cDNA was prepared using an iscript cDNA synthesis kit (BioRad Laboratories, USA). Quantitative real-time PCR was performed using SSO-Advanced Universal SYBR Green supermix (BioRad Laboratories, USA) and was analyzed on the CFX384 Touch real-time PCR system (BioRad Laboratories). Transcript levels were measured using the Delta-Ct method after normalization to housekeeping genes (race paper)

Olink Assay

Serum cytokines were analyzed using the Olink multiplex assay platform (Immuno-oncology Panel, Olink Bioscience), according to the manufacturer's instructions.

Briefly, an incubation master mix containing pairs of oligonucleotide-labeled antibodies to each protein was added to the samples and incubated for 16 h at 4 ºC. Each protein was targeted with two different epitope-specific antibodies increasing the specificity of the assay. The presence of the target protein in the sample brought the partner probes in proximity, allowing the formation of a double-strand oligonucleotide polymerase chain reaction (PCR) target. On the following day, the extension master mix in the sample initiated the specific target sequences to be detected and generated amplicons using PCR in 96 well plates. For the detection of the specific protein, a Dynamic array integrated fluidic Circuit (IFC) 96 × 96 chip was primed, loaded with 92 protein-specific primers, and mixed with sample amplicons including three inter-plate controls (IPS) and three negative controls (NC). Real-time microfluidic qPCR was performed in Biomark (Fluidigm, San Francisco, CA) for the target protein quantification.

Analysis of O-Link data

Data analysis was done using real-time PCR analysis software (ΔΔCt method [doi:10/c689hx] and NPX (Normalized Protein Expression) manager. For data normalization, internal controls were used in each sample, inter-plate control (IPC) and negative controls, and a correction factor was calculated by Olink from the negative controls. This produces NPX values that are proportional to log2 of the protein concentration. Clustergrammer [23] was used to visualize the expression of the target proteins.

In multivariate analysis, the genes were grouped according to their pathways to create corresponding models when using genes from that pathway alone, and in pooled approach where all genes were grouped together in the joint multivariate model. Genes were selected using the Maximum Relevance Minimum Redundancy (mRMR) technique to select a subset of best genes having the most correlation with class characterization (relevance), and the smallest correlation between each other (redundancy). Groups of genes were compared with the F-test of corresponding logistic regression models to see if a more complex model provided a significantly better fit to the observations than the corresponding model with fewer parameters. The ability of the models to predict inflammation was evaluated by the ROC curve analysis using AUC values. All statistical calculations were performed with Rstudio 1.1.456 (Rstudio Inc.).

Table S1. Subgroup multivariable analysis predicting adverse pathology considering pelvic inflammation without hernia mesh in 1858 cases.

Covariates.	OR (CI 95%)	*p* Value
PSA (ng/mL)	2.376 (1.774, 3.183)	<0.0001
Biopsy Gleason		
1,2	Ref	
3	4.941 (3.801, 6.423)	0.5929
4,5	20.981 (14.801, 29.740)	<.0001
herniamesh_pelvicinflammation		
absent	Ref	
present	1.320 (1.033, 1.685)	0.0262

Table S2. Subgroup multivariable analysis predicting adverse pathology in high Biopsy Gleason group (3,4,5) in 881 cases.

Covariates	Odds Ratio (95% CI)	*p* Value
PSA	1.90 (1.28,2.82)	0.0014
Pelvic Inflammation	1.45 (1.07, 1.99)	0.0185
ECE on MRI	2.60 (1.85, 3.67)	<0.0001

Table S3. Univariable and Multivariable analysis predicting Adverse Pathology considering pelvic inflammation.

	Univariable analysis		Multivariable Analysis	
Covariate	Odds ratio ((5% CI)	*p* Value	Odds ratio ((5% CI)	*p* Value
Age (years)	1.06 (1.05, 1.07)	<0.0001	1.06(1.05, 1.08)	<0.0001
PSA (ng/mL)				
<10 ng/mL	Ref		Ref	
>10.1 ng/mL	2.87 (2.27, 3.62)	<0.0001	3.11 (2.43, 3.98)	<0.0001
Race				
African American	0.69 (0.47, 1.03)	0.0108		
Caucasian	0.97 (0.71, 1.32)	0.6654		
Asian	1.15 (0.72, 1.85)	0.1778		
Others	Ref			
MRI Prostate Volume	0.99 (0.99, 1.00)	0.2762	0.99 (0.99, 0.99)	<0.0001
Pelvic Inflammation				
Low (0,1)	Ref		Ref	
High (2,3)	1.69 (1.41, 2.02)	<0.0001	1.51 (1.25, 1.82)	<0.0001
Herniamesh_pelvicinflam				
No inflammation w/o hernia mesh	Ref			
Inflammation without hernia mesh	1.81 (1.48, 2.22)	0.0002		
Inflammation with hernia mesh	1.45 (1.10, 1.91)	0.5911		
MRI ECE				
Absent	Ref			
present	2.98 (2.44, 3.64)	<0.0001		
MRI PI-RADS		<0.0001		
1/2/3	Ref			
4/5	2.43 (1.95, 3.03)			
Biopsy Gleason Grade Group		<0.0001		
1/2	Ref			
3/4/5	10.185 (8.272, 12.53)			

Table S4. Baseline characteristics between patients with or without Biochemical Recurrence and PSA persistence features.

Covariates	BCR Absent (*n*= 1719)	BCR present (*n*= 278)	*p* Value
Age (years)	63.0 (57.1, 68.0)	65.0 (60.2,69.2)	<0.0001
PSA (ng/mL)	5.5 (4.4, 8.2)	8.5 (5.6, 16.0)	<0.0001
MRI prostate Volume (mL)	37 (28.0, 52.0)	38 (27.0, 54.0)	0.7531
Clinical Stage			<0.0001
T1	817 (6675%)	115(54.50%)	
T2	393 (32.11%)	82 (38.86%)	
T3	14 (1.14%)	14 (6.64%)	
Pelvic Inflammation			0.2687
0 (absent)	426 (24.78%)	76 (27.34%)	
1 (low)	611 (35.54%)	83 (29.85%)	
2 (moderate)	327 (19.02%)	53 (19.06%)	
3 (high)	355 (20.65%)	66 (23.74%)	
Race			0.1168
African American	189 (10.99%)	43 (16.94%)	
Caucasian	1282 (74.58%)	190 (68.35%)	
Asian	92 (5.35%)	17 (6.12%)	
other	156 (9.08%)	28 (10.07%)	
MRI ECE			<0.0001
Absent	1263 (72.65%)	136 (48.92%)	
Present	456 (26.53%)	142 (51.08%)	
MRI_PIRADS			<0.0001
0	44 (2.56%)	4 (1.44%)	
1	83 (4.83%)	18 (6.47%)	
2	177 (10.30%)	5 (1.80%)	
3	146 (8.49%)	10 (3.6%)	
4	773 (44.97%)	74 (26.62%)	
5	496 (28.85%)	167 (60.07%)	
Biopsy Gleason Grade			<0.0001
3+3	385 (22.40%)	16 (5.76%)	
3+4	706 (41.07%)	52 (18.71%)	
4+3	323 (18.79%)	74 (26.62%)	
4+4/3+5/5+3	192 (11.17%)	73 (26.26%)	
4+5/5+4/5+5	113 (6.57%)	63 (22.66%)	
Path Gleason grade Group			<0.0001
3+3	214 (12.45%)	3 (1.08%)	
3+4	1024 (59.57%)	66 (24.79%)	
4+3	359 (20.88%)	125 (44.96%)	
4+4/3+5/5+3	34 (1.98%)	18 (6.47%)	
4+5/5+4/5+5	88 (5.12%)	66 (23.74%)	
Pathology EPE			<0.0001
absent	1403 (81.62%)	127 (45.68%)	
present	316 (18.38%)	151 (54.32%)	
Seminal Vesicular Invasion			<0.0001
absent	1609 (93.60%)	188 (67.63%)	
present	110 (6.40%)	90 (32.37%)	
Positive Surgical Margin			<0.0001
absent	1613 (93.83%)	232 (83.45%)	
present	106 (6.17%)	46 (16.55%)	
Lymphovacular invasion			<0.0001

absent	1645 (97.05%)	226 (81.29%)	
present	50 (2.95%)	52 (18.71%)	
Final Pathology Stage			<0.0001
pT2	468 (27.23%)	29 (10.43%)	
pT2a	130 (7.56%)	9 (3.24%)	
pT2b	8 (0.47%)	3 (1.08%)	
pT2c	766 (44.56%)	71 (25.54%)	
pT3a	240 (13.96%)	76 (27.34%)	
pT3b	107 (6.22%)	90 (32.37%)	
Herniamesh_pelvic inflammation			0.4047
No inflammation w/o hernia mesh	1037 (60.33%)	159 (57.19%)	
Inflammation without hernia mesh	465 (27.05%)	86 (30.94%)	
Inflammation with hernia mesh	217 (12.62%)	33 (11.87%)	

Table S5. Cox regression models for preoperative and postoperative predictors of BCR and PSA persistence after RP in 1997 patients.

Univariate			Multivariable	
**Covariates**	**HR (95% CI)**	***p* Value**		
Age (years)	1.04(1.03, 1.06)	<0.0001		
PSA (ng/mL)				
<10 ng/mL	Ref			
>10.1 ng/mL	3.85 (3.03, 4.90)	<.0001		
Race				
African American	1.22 (0.75, 1.97)	0.4241		
Caucasian	0.72 (0.48, 1.08)	0.1083		
Asian	0.92 (0.50, 1.69)	0.7870		
Others	Ref			
MRI Prostate Volume (mL)	1.002 (0.99, 1.01)	0.2334		
Pelvic Inflammation				
Low (0,1)	Ref			
High (2,3)	1.48(1.16, 1.89)	0.0014	1.29 (1.01, 1.64)	0.0433
MRI ECE				
Absent	Ref			
present	2.45 (1.93, 3.09)	<.0001		
MRI PI-RADS				
1/2/3	Ref			
4/5	2.34 (1.66, 3.32)	<0.0001		
Biopsy Gleason Grade Group				
1/2	Ref			
3/4/5	4.93 (3.75, 6.49)	<.0001		
Path Gleason Grade group				
1,2	Ref			
3,4,5	6.45 (4.91, 8.46)	<.0001		
Positive surgical margin				
Absent	Ref			
Present	2.41 (1.76, 3.32)	<.0001	1.40 (1.01, 1.94)	0.0454
Pathology Stage				
pT2	Ref			
pT3	4.25 (3.34, 5.4)	<0.0001	3.94 (3.07, 5.05)	<0.0001
Lymph Node invasion				
Absent	Ref			
Present	4.48 (3.31, 6.07)	<.0001		
SVI				
Absent	Ref			
Present	4.37 (3.39, 5.62)	<.0001		

EPE		
Absent	Ref	
Present	3.84 (3.03, 4.86)	<.0001

Table S6. Cox regression models for predictors including pT3 Stage, pathology stage and pelvic inflammation for predicting BCR after RP in 1997 patients.

**Covariates**	**HR (95 % CI)**	***p* Value**
Pelvic Inflammation		
0,1 (low)		
2,3 (high)	1.32 (1.03, 1.68)	0.0274
PSA		
<10 ng/mL		
>10.1 ng/mL	2.81 (2.19, 3.60)	<0.0001
Pathology Stage		
pT2		
pT3	3.36 (2.62, 4.30)	<0.0001

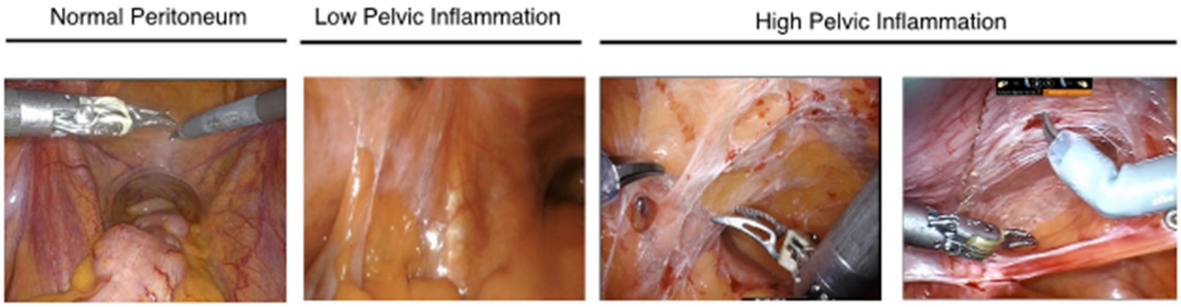

Figure S1. Pelvic Inflammation (VISUAL GRADING).

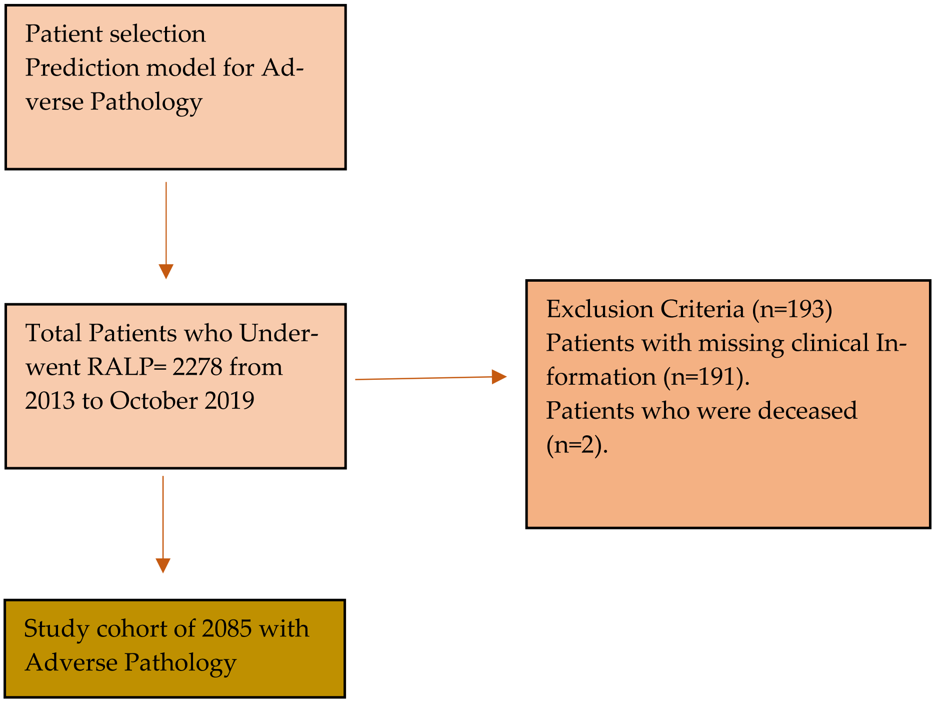

Figure S2. CONSORT flow diagram of study cohort forAdverse Pathology endpoint

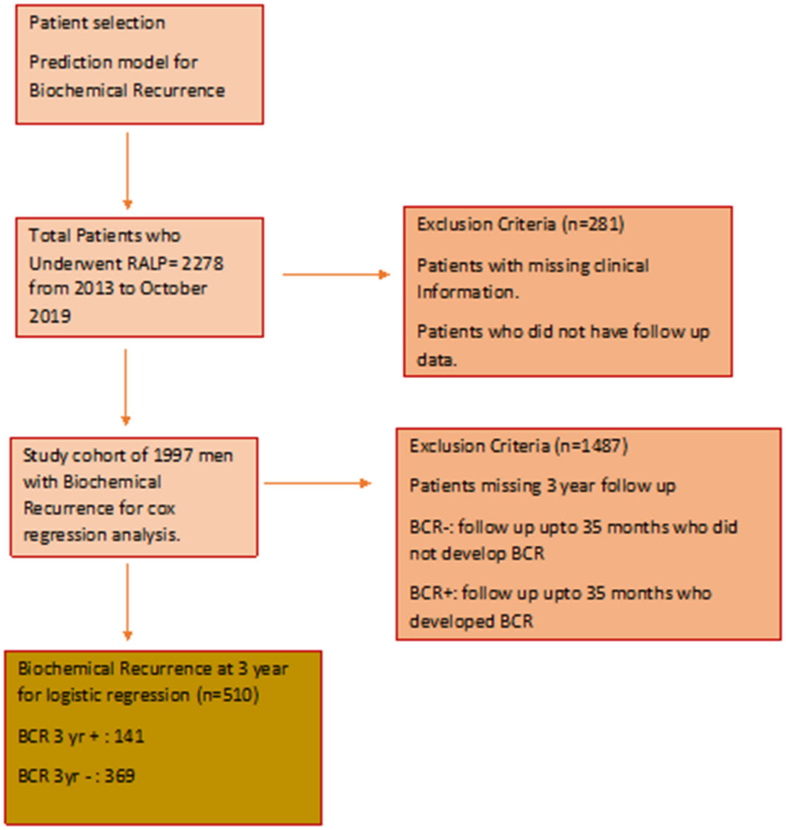

Figure S3. CONSORT flow diagram of study cohort for Biochemical Recurrence and PSA Persistence.

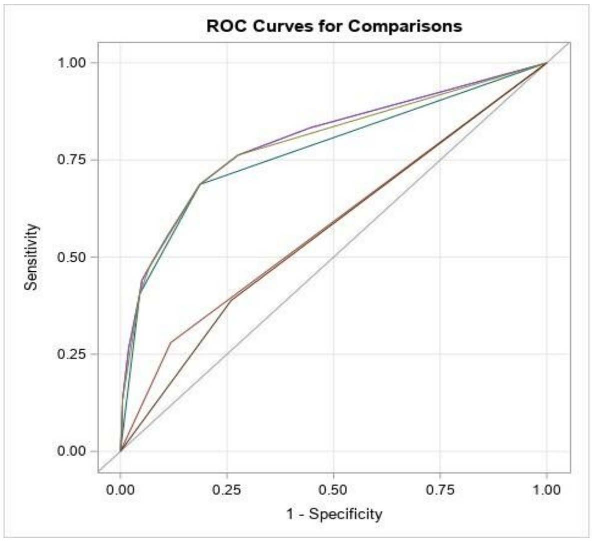

**ROC Contrast Estimation and Testing ResuIts by Row**						
**Contrast**	Estimate	Standard Error	95% Wald Confidence Limits		Chi-Square	Pr > ChiSq
**modeI without infIammation- modeI with infIammation and without hernia**	−0.00892	0.00371	−0.0162	−0.00165	5.7774	0.0162

Figure S4. Area under receiver operating characteristics for prediction of Adverse Pathology in subgroup considering pelvic inflammation without hernia mesh. [a] Under the nonparametric assumption. [b] Null hypothesis: true area= 0.05

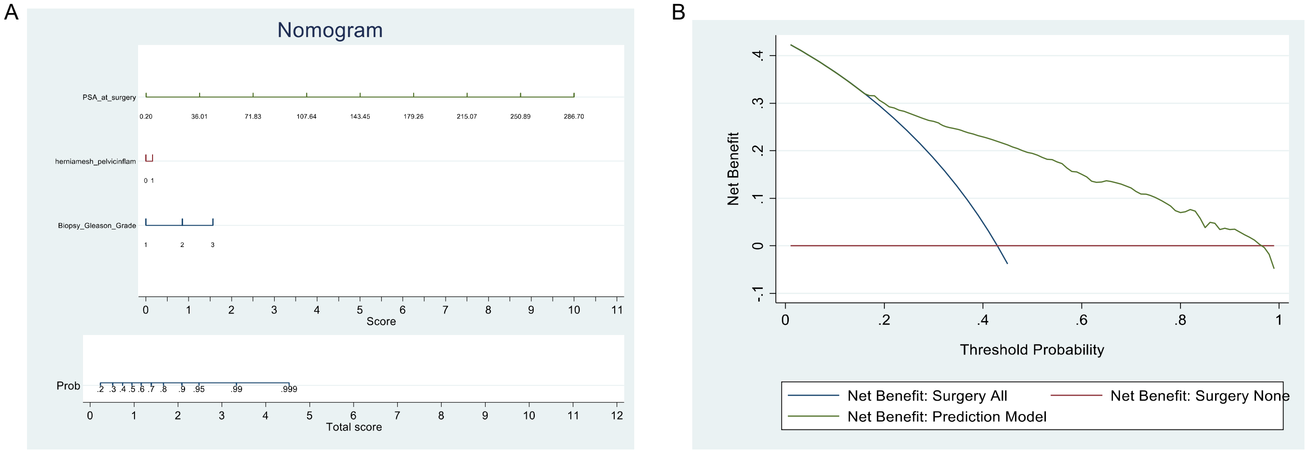

Figure S5. (**A**). Nomogram built for the prediction of Adverse Pathology in the subgroup cohort of 1858 cases. (**B**). Decision curve analysis for predicting AP using prediction model based on subgroup analysis. The unit is the benefit associated with one PCa patient duly undergoing surgery.

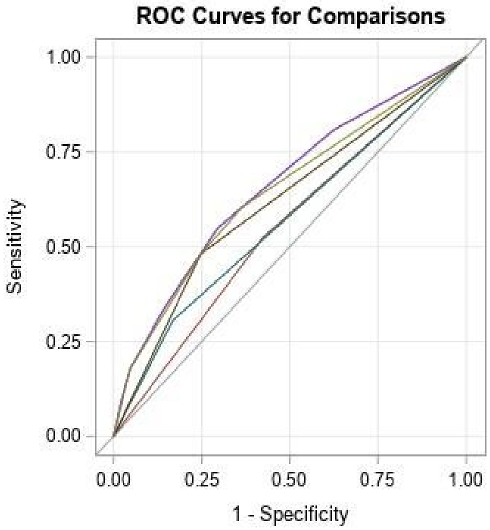

ROC Model	Area
**Model**	0.6593
**Presence of pelvic_Inflammation (0/1vs 2/3)**	0.5502
**PSA**	0.5686
**ECE on mpMRI**	0.6166
**with pelvic_inflammation**	0.6593
**without pelvic_inflammation**	0.6433

ROC Contrast Estimation and Testing Results by Row						
Contrast	Estimate	Standard Error	95% Wald Confidence Limits		Chi-Square	Pr > ChiSq
without infl − with infl	−0.0161	0.00810	−0.0319	−0.00018	3.9311	0.0474

Figure S6. Area under receiver operating characteristics for prediction of Adverse Pathology considering pelvic inflammation in high biopsy Gleason grade group (3,4,5) in 881 patients.

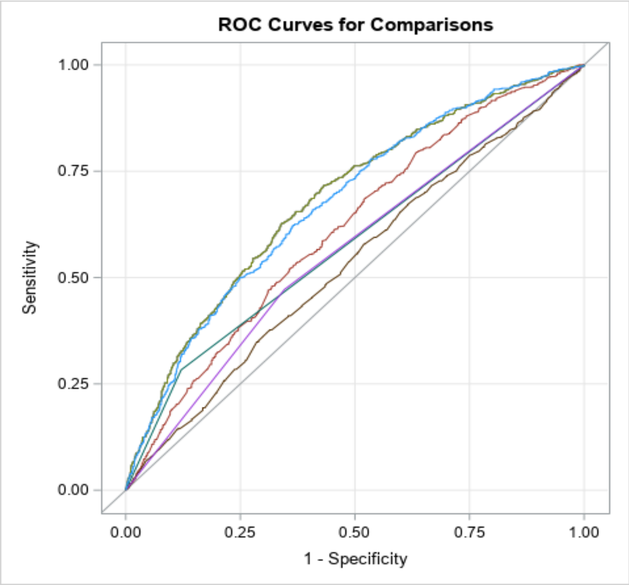

ROC Model	Area Under Curve
Model	0.6840
Age	0.6126
PSA	0.5810
Prostate_Volume	0.5329
Presence of pelvic inflammation (0/1 vs 2/3)	0.5629
model with pelvic inflammation	0.6840
model without pelvic inflammation	0.6750

ROC Contrast Estimation and Testing Results by Row						
Contrast	Estimate	Standard Error	95% Wald Confidence Limits		Chi-Square	Pr > ChiSq
Model without pelvic inflammation-model with pelvic inflammation	-0.00998	0.00446	-0.0187	-0.00124	5.0053	0.0253

Under the nonparametric assumption
Null hypothesis: true area= 0.05

Figure S7. Area under receiver operating characteristics for prediction of Adverse Pathology considering pelvic inflammation

Tine after surgery (months)	0	20	40	60	80
**Low Pelvic Inflammation**	1196	493	221	56	1
**High Pelvic Inflammation**	801	263	59	7	0

Figure S8. Risk Table: Numbers of survival probability of persons at risk at the beginning of the period of follow-up after Radical Prostatectomy by pelvic inflammation

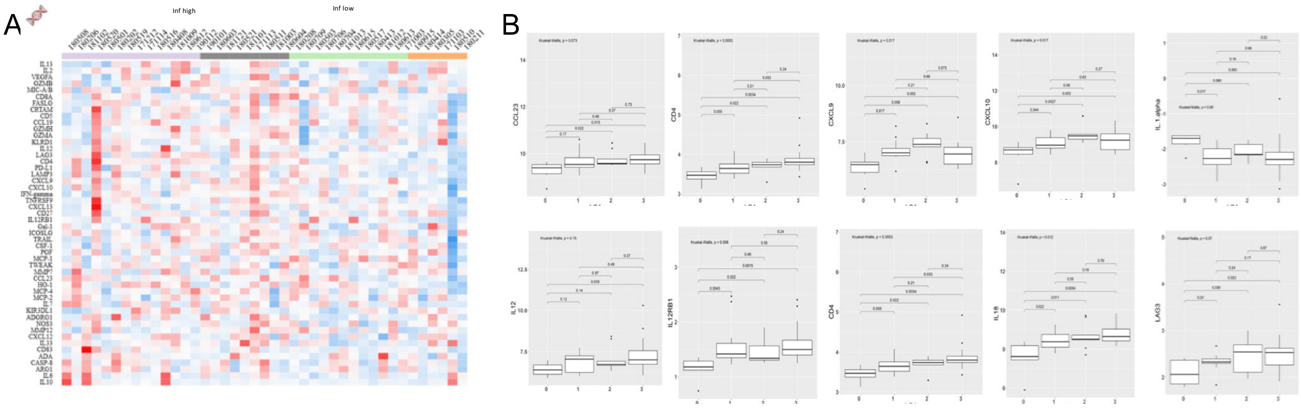

Figure S9. (**A**). Levels of 92 immune-oncology markers were evaluated in the serum of PCa patients with or without pelvic inflammation using O-link and heat map of differentially expressed genes between the inflammation groups is shown ($p < 0.05$). (**B**). Proteins/cytokines differentially expressed in different pelvic inflammation groups; Pelvic inflammation score: 0-3

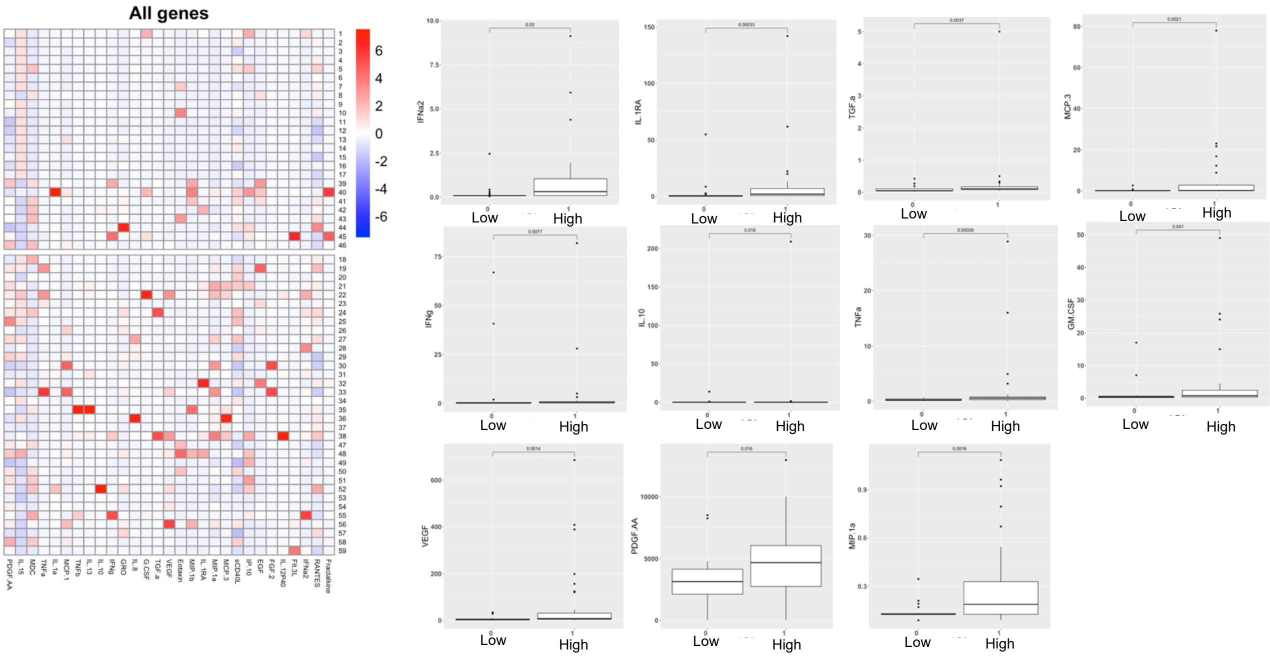

Figure S10. Proteins/cytokines differentially expressed in different pelvic inflammation groups; Pelvic inflammation score: 0-3

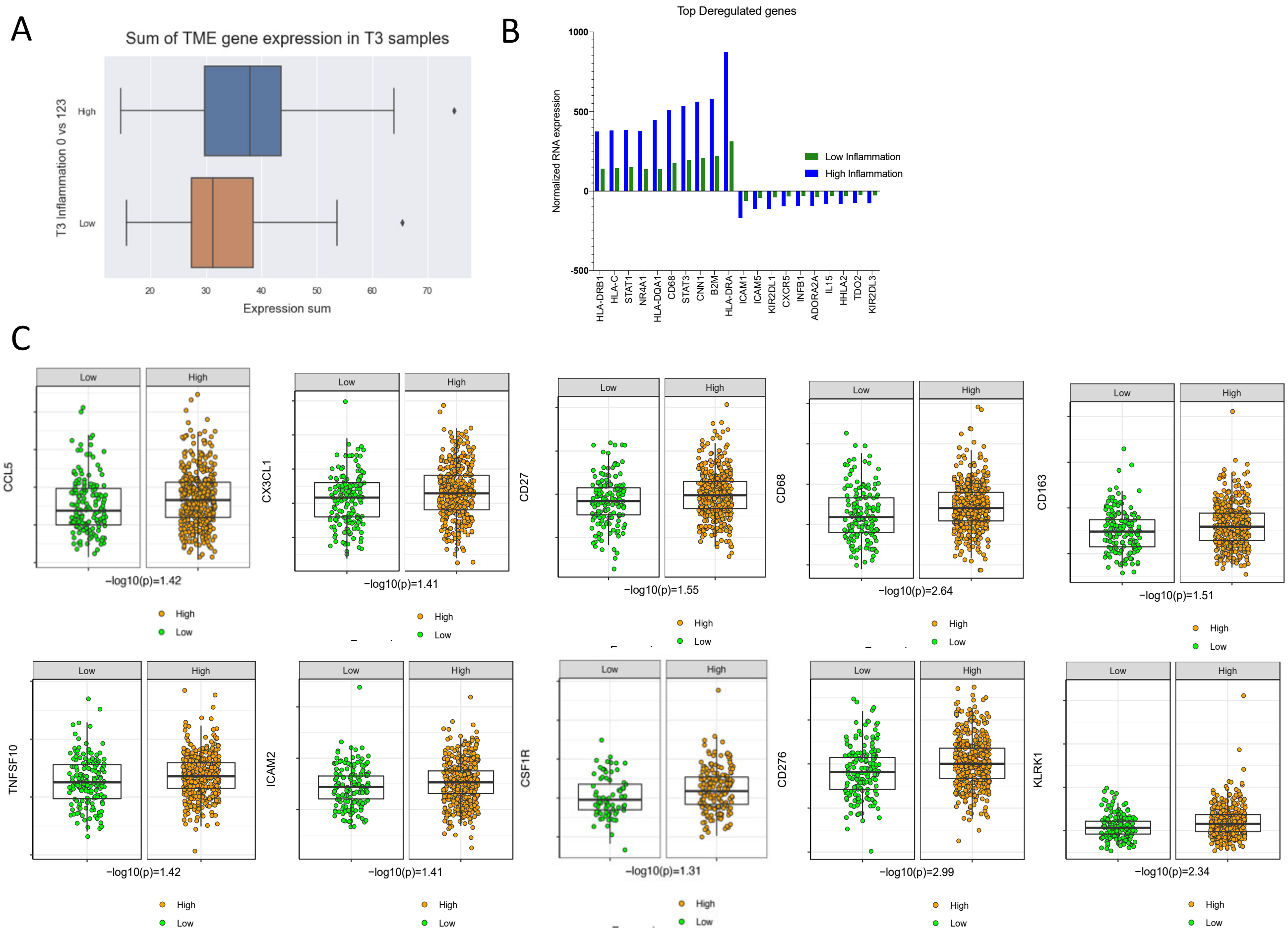

Figure S11. Prostate cancer patients with pelvic inflammation demonstrate elevated levels of inflammatory genes. A. Analysis of 146 genes in prostate cancer patients with T2 and T3 disease and pelvic inflammation. B. Genes significantly deregulated in patients with high inflammation and T3 disease. C. Expression of proinflammatory cytokines in tissue from prostate cancer patients with low and high pelvic inflammation.

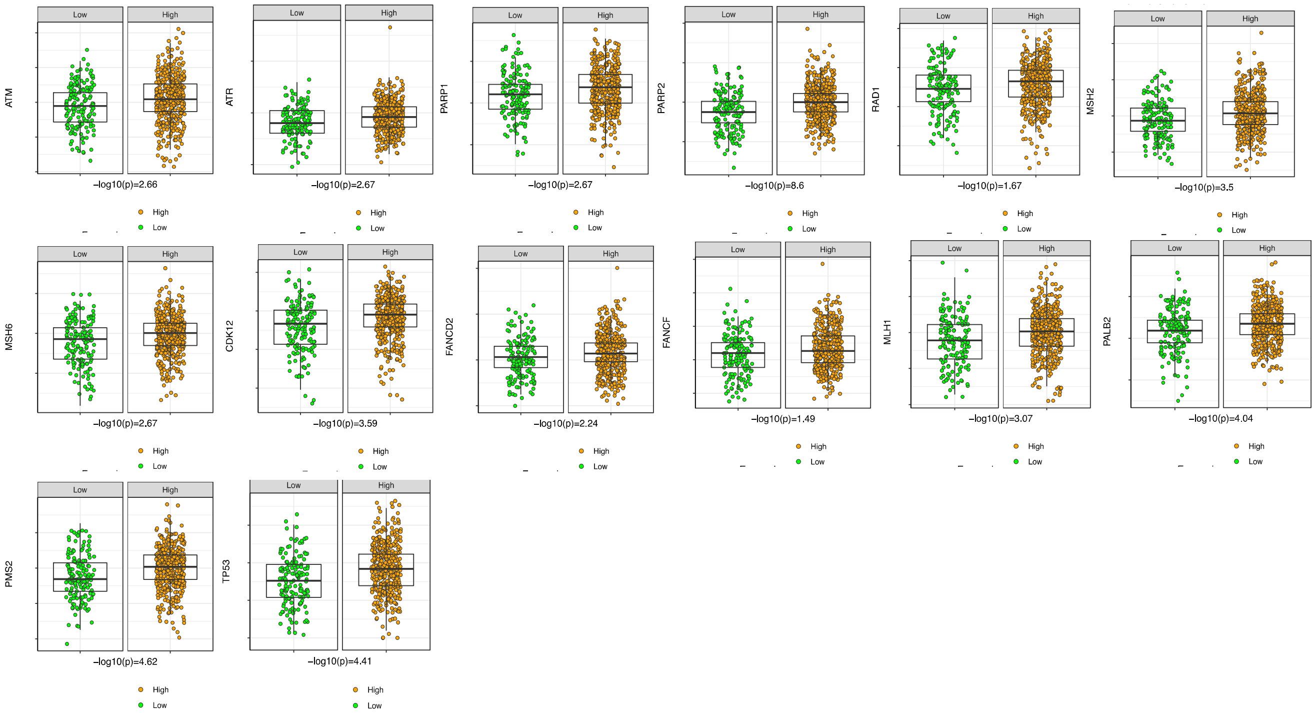

Figure S12. Prostate cancer patients with pelvic inflammation demonstrate elevated levels of DDR genes. DDR genes are significantly upregulated in prostate cancer from patients with high pelvic inflammation

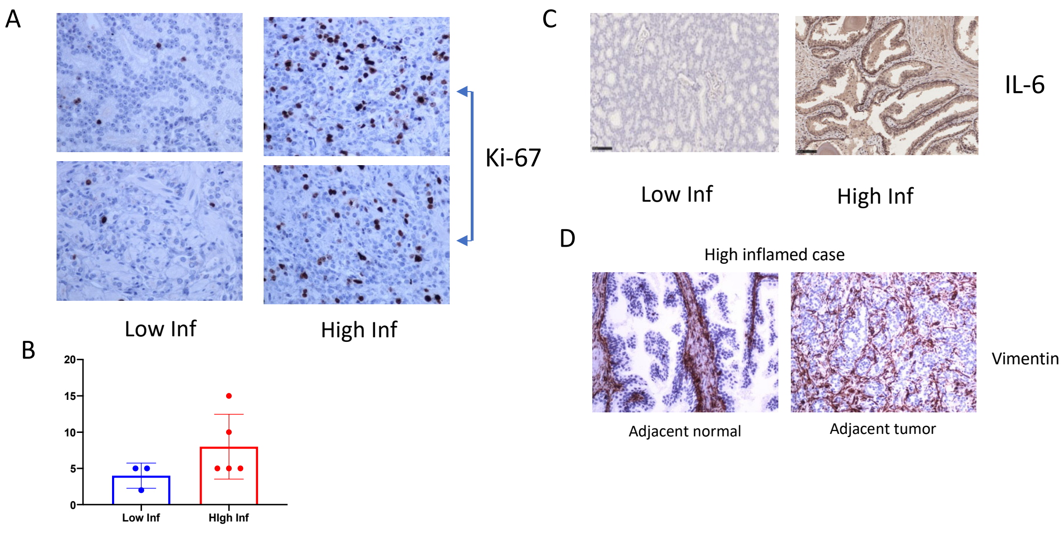

Figure S13. Prostate tissues of prostate cancer patients with high pelvic inflammation shows increased proliferation and expression of proinflammatory cytokines. (**A**,**B**). Ki-67 staining in PCa tissue from patients with low and high pelvic inflammation. (**C**). IL-6 expression in prostate cancer tissues of patients with low and high inflammation. (**D**). Vimentin expression in prostate tissue of prostate cancer tissue patents with high pelvic inflammation. Magnification 20×.
